# Association Between Inflammatory Bowel Disease and Venous Leg Ulcers: Insight From Mendelian Randomization Analyses

**DOI:** 10.1155/cjgh/6767725

**Published:** 2026-01-21

**Authors:** Yanfeng Lin, Xiaohui Qin, Haiyan Zhang, Jinke Huang

**Affiliations:** ^1^ Department of Gastroenterology, The Second Affiliated Hospital of Guangzhou University of Chinese Medicine (Guangdong Provincial Hospital of Chinese Medicine), Guangzhou, China, gzucm.edu.cn; ^2^ Department of Neurology, The Second Affiliated Hospital of Guangzhou University of Chinese Medicine (Guangdong Provincial Hospital of Chinese Medicine), Guangzhou, China, gzucm.edu.cn

**Keywords:** Crohn’s disease, inflammatory bowel disease, ulcerative colitis, venous leg ulcers

## Abstract

**Background:**

This study aimed to investigate the potential causal relationship between inflammatory bowel disease (IBD) and venous leg ulcers (VLU) using Mendelian randomization (MR).

**Materials and Methods:**

Independent genetic variants for IBD and VLU were selected as instruments from previously published genome‐wide association studies (GWAS) in people of primarily European descent. MR analyses were carried out utilizing inverse‐variance weighted (IVW), weighted median, MR Egger, simple mode, and weighted mode.

**Results:**

Genetically predicted IBD was not associated with VLU, according to the IVW analysis (OR 0.95, 95% CI 0.88–1.03, *p* = 0.23). MR Egger (OR 0.93, 95% CI 0.76–1.12, *p* = 0.44), weighted mode (OR 1.06, 95% CI 0.87–1.29, *p* = 0.55), simple mode (OR 1.03, 95% CI 0.79–1.34, *p* = 0.83), and weighted median (OR 0.99, 95% CI 0.89–1.10, *p* = 0.90) all produced results in line with IVW. According to the IVW technique, UC (OR 1.02, 95% CI 0.95–1.10, *p* = 0.62) and CD (OR 0.97, 95% CI 0.90–1.04, *p* = 0.36) did not appear to be associated with VLU in subtype analyses.

**Conclusion:**

The MR investigation found no genetic evidence to support a causal relationship between IBD and VLU. This finding clarifies that observed clinical associations are unlikely to be driven by shared genetics, and this genetic insight helps refine the clinical assessment of VLU in patients with IBD.

## 1. Introduction

Inflammatory bowel disease (IBD), encompassing ulcerative colitis (UC) and Crohn’s disease (CD), is a chronic, immune‐mediated disorder of the gastrointestinal tract characterized by relapsing inflammation [1]. While CD features discontinuous transmural lesions, UC typically presents with continuous inflammation confined to the mucosa [2]. The global incidence of IBD is rising, imposing a substantial burden on healthcare systems and patient quality of life [3–6].

Venous leg ulcers (VLU), chronic wounds primarily caused by venous insufficiency, affect a significant proportion of the elderly population, with a prevalence of up to 5% in those aged over 65 [7, 8]. Notably, cases of VLU in IBD patients are frequently reported [9, 10], suggesting a potential pathophysiological link. This association may be driven by systemic manifestations of IBD, including chronic inflammation that promotes endothelial dysfunction and a well‐documented hypercoagulable state [11, 12]. Both mechanisms are implicated in venous stasis and vascular injury, key processes in VLU pathogenesis. However, whether this relationship is causal remains uncertain due to confounding factors in observational studies. Therefore, a biological rationale supports investigating a potential causal link between these conditions.

In this study, we employed Mendelian randomization (MR), a genetic epidemiological approach that uses genetic variants as instrumental variables to infer causality [13]. As genetic alleles are randomly allocated at conception, MR minimizes confounding by environmental factors and reverse causality. This study aims to elucidate the potential causal relationship between IBD (and its subtypes) and VLU using this robust methodology.

## 2. Materials and Methods

### 2.1. Summary Data Source

To adequately establish causal effects, the single‐nucleotide polymorphisms (SNPs) used in the MR study must satisfy the following key hypotheses: (a) SNPs and exposure must be substantially correlated; (b) SNPs have to be independent of any factors linked to the outcome; (c) Only through the specified exposure may SNPs be connected to outcomes; no other means are acceptable. A GWAS with European participants yielded summary statistics on IBD, covering the disease entities UC (33,977 controls, 13,768 cases) and CD (33,977 controls, 17,897 cases), as well as IBD overall (33,977 controls, 31,655 cases). The summary data for VLU were derived from a GWAS comprising 1584 patients and 207,482 controls of European ancestry. Details of the included datasets are presented in Table 1.

**Table 1:** Basic information of the included datasets.

Disease	ID	Controls	Cases	No. of SNPs
VLU	finn‐bL12_ULCERLOWLIMB	207,482	1584	16,380,446
IBD	ieu‐a‐294	33,977	31,665	157,116
UC	ieu‐a‐970	33,977	13,768	156,116
CD	ieu‐a‐12	33,977	17,897	124,888

### 2.2. Selection of Instrumental Variables

Given their critical importance to maintaining the validity of the study, the following procedure was employed to select the best instrumental variables. To be used as genetic tools in the MR analyses, SNPs were first selected at the genome‐wide significance criterion of *p* < 5 × 10^−8^. A linkage disequilibrium clumping technique was used, with the stringent cut‐off values of *r* ^2^ = 0.001 and distance = 10000 kb, to guarantee the independence of the instruments. After screening, 120, 86, and 130 SNPs associated with overall exposure to CD, UC, and IBD were finally selected for MR analysis.

### 2.3. Statistical Analyses

Using the online analytic platform (http://app.mrbase.org/), the “TwoSampleMR” R package was used to conduct the MR analyses. The core MR analysis was conducted using the usual inverse variance weighted model (IVW) [14]. Causal effects were also estimated using the weighted median, MR Egger, and weighted mode. The leave‐one‐out strategy was used to conduct the sensitivity analysis. The analysis results were presented using forest plots, funnel plots, and scatter plots. *p* < 0.05 was statistically significant as an indicator of a potential causal influence.

## 3. Results

### 3.1. MR Analysis for Causal Link of IBD With VLU

**Figure 1:**
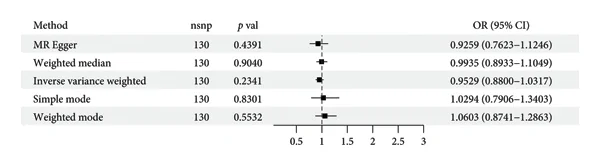
Causal estimates were presented using odds ratios (ORs) and 95% confidence intervals (CIs) for the effects of IBD on VLU.

**Figure 2:**
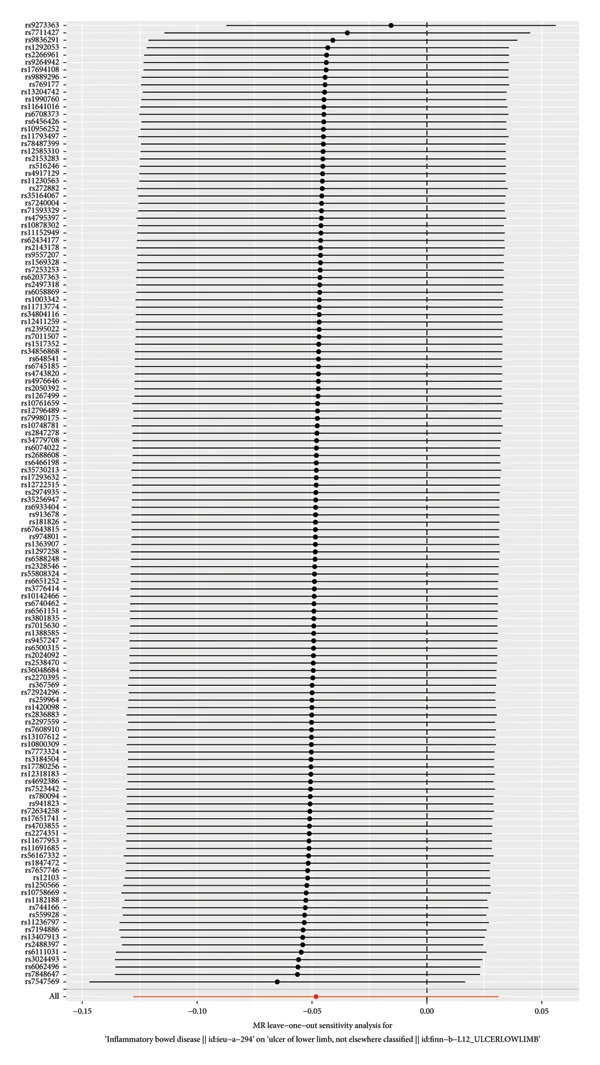
Forest plots depicting the leave‐one‐out analysis of the causal relationships between IBD and VLU.

**Figure 3:**
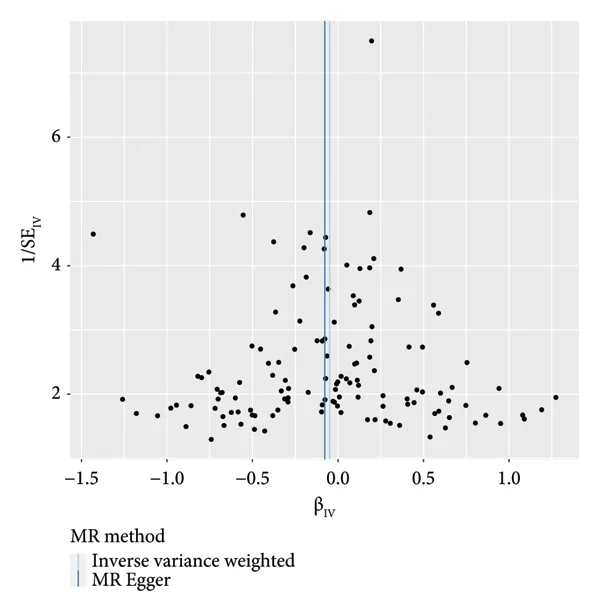
Funnel plots for this MR analysis of the causal relationship between IBD and VLU.

The MR estimates from the various models used to assess how IBD and VLU are causally related are displayed in Figure 1. The IVW analysis revealed that genetically predicted IBD was not linked to VLU (OR 0.95, 95% CI 0.88–1.03, *p* = 0.23). Models of simple mode (OR 1.03, 95% CI 0.79–1.34, *p* = 0.83), weighted mode (OR 1.06, 95% CI 0.87–1.29, *p* = 0.55), weighted median (OR 0.99, 95% CI 0.89–1.10, *p* = 0.90), and MR Egger (OR 0.93, 95% CI 0.76–1.12, *p* = 0.44) yielded results consistent with IVW. Although Cochran’s Q test indicated the presence of heterogeneity (*p* < 0.05), which can be a sign of pleiotropy, the MR‐Egger intercept test did not detect significant horizontal pleiotropy (*p* > 0.05) (Table 2). The robustness of this null association was further supported by leave‐one‐out sensitivity analysis (Figure 2), which confirmed that no individual SNP was exerting a disproportionate influence on the overall result. Additionally, visual inspection of the funnel plot (Figure 3) showed no substantial asymmetry, reducing concerns about directional pleiotropy.

**Table 2:** Results of sensitivity analysis using different analytical methods.

Exposure	Outcome	MR methods	Cochran *Q* statistic	Heterogeneity *p* value	Pleiotropy *p* value	PRESSO global outlier test	
						RSSOBs	*p* value
IBD	VLU	IVW	185.4639	0.0008	0.7514	189.9097	4*e* − 04
UC	VLU	IVW	111.3225	0.0751	0.7136	115.0271	0.0252
CD	VLU	IVW	170.8689	0.0013	0.4739	173.6894	8*e* − 04

### 3.2. MR Analysis for Causal Link of UC With VLU

**Figure 4:**
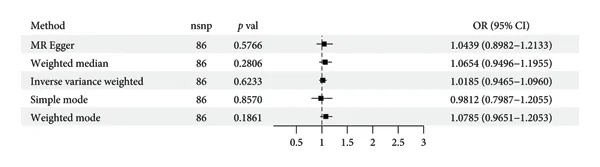
Causal estimates were presented using odds ratios (ORs) and 95% confidence intervals (CIs) for the effects of UC on VLU.

**Figure 5:**
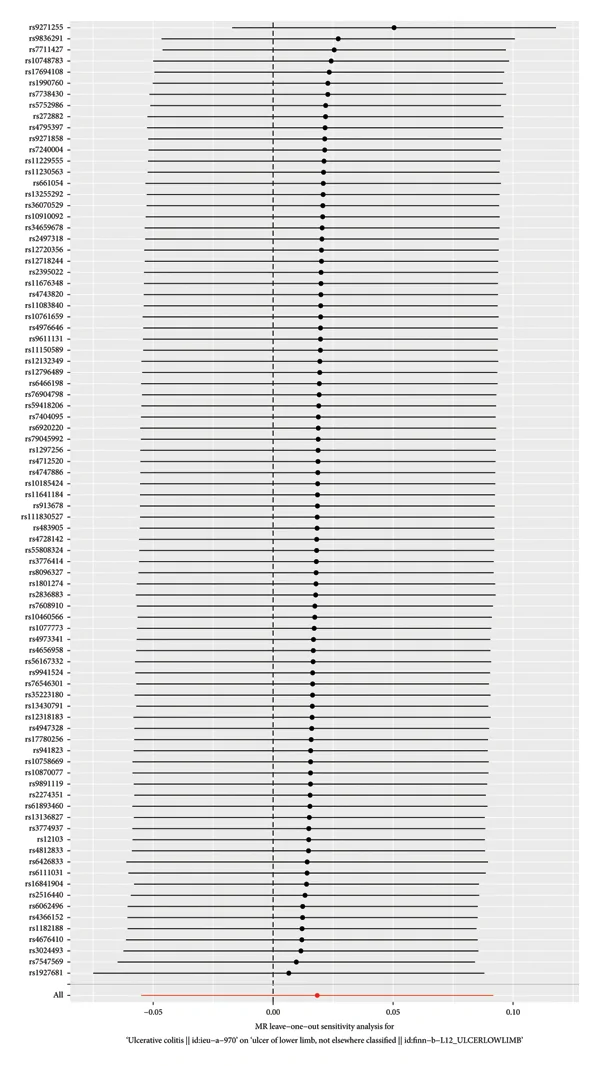
Forest plots depicting the leave‐one‐out analysis of the causal relationships between UC and VLU.

**Figure 6:**
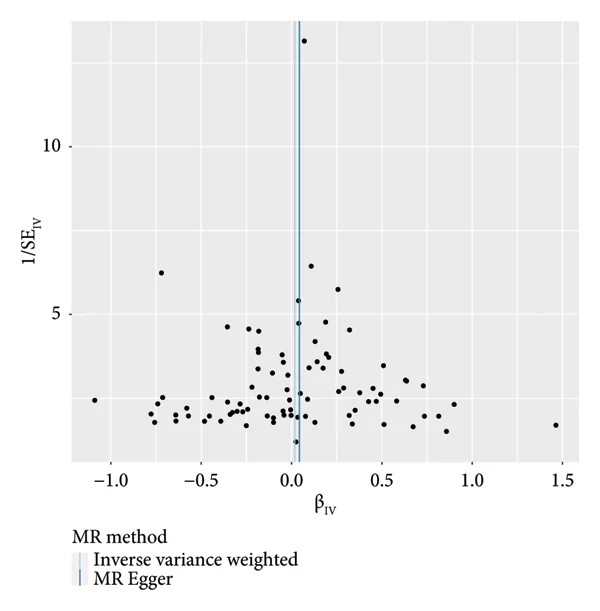
Funnel plots for this MR analysis of the causal relationship between UC and VLU.

When UC and CD were considered separately, the IVW analysis (OR 1.02, 95% CI 0.95–1.10, *p* = 0.62) revealed that genetically predicted UC was not linked to VLU (Figure 4). Models of simple mode (OR 0.98, 95% CI 0.80–1.21, *p* = 0.86), weighted mode (OR 1.08, 95% CI 0.97–1.21, *p* = 0.19), weighted median (OR 1.07, 95% CI 0.95–1.20, *p* = 0.28), and MR Egger (OR 1.04, 95% CI 0.90–1.21, *p* = 0.58) yielded results consistent with IVW. Although Cochran’s Q test indicated the presence of heterogeneity (*p* < 0.05), which can be a sign of pleiotropy, the MR‐Egger intercept test did not detect significant horizontal pleiotropy (*p* = 0.75). The robustness of this null association was further supported by leave‐one‐out sensitivity analysis (Figure 5), which confirmed that no individual SNP was exerting a disproportionate influence on the overall result. Additionally, visual inspection of the funnel plot (Figure 6) showed no substantial asymmetry, reducing concerns about directional pleiotropy.

### 3.3. MR Analysis for Causal Link of CD With VLU

**Figure 7:**
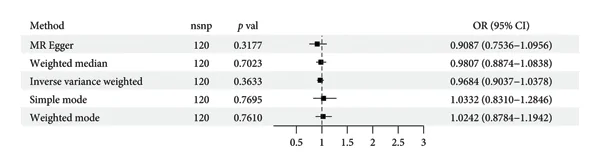
Causal estimates were presented using odds ratios (ORs) and 95% confidence intervals (CIs) for the effects of CD on VLU.

**Figure 8:**
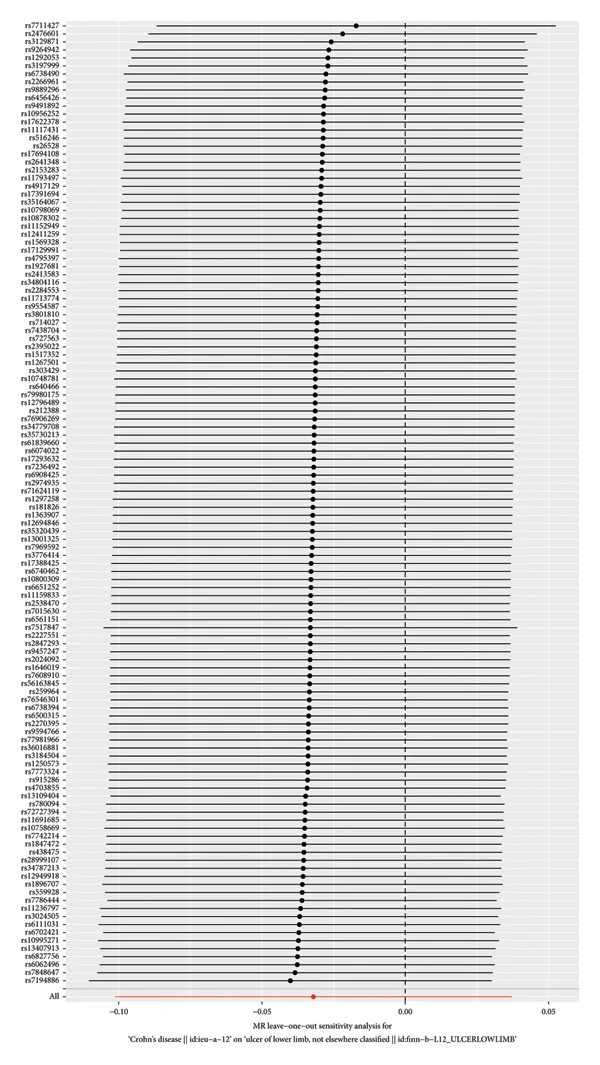
Forest plots depicting the leave‐one‐out analysis of the causal relationships between CD and VLU.

**Figure 9:**
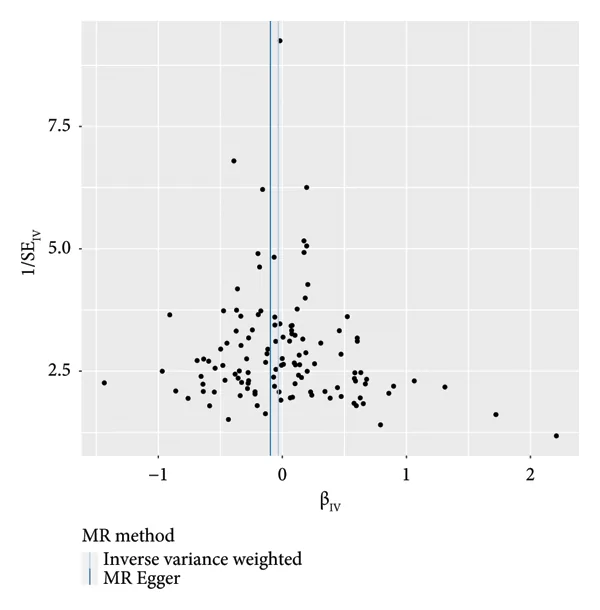
Funnel plots for this MR analysis of the causal relationship between CD and VLU.

When UC and CD were considered separately, the IVW analysis (OR 0.97, 95% CI 0.90–1.04, *p* = 0.3633) revealed that genetically predicted CD was not linked to VLU (Figure 7). Models of simple mode (OR 1.03, 95% CI 0.83–1.28, *p* = 0.77), weighted mode (OR 1.02, 95% CI 0.88–1.19, *p* = 0.76), weighted median (OR 0.98, 95% CI 0.89–1.08, *p* = 0.70), and MR Egger (OR 0.91, 95% CI 0.76–1.10, *p* = 0.32) yielded results consistent with IVW. Although Cochran’s Q test indicated the presence of heterogeneity (*p* < 0.05), which can be a sign of pleiotropy, the MR‐Egger intercept test did not detect significant horizontal pleiotropy (*p* > 0.05) (Table 2). The robustness of this null association was further supported by leave‐one‐out sensitivity analysis (Figure 8), which confirmed that no individual SNP was exerting a disproportionate influence on the overall result. Additionally, visual inspection of the funnel plot (Figure 9) showed no substantial asymmetry, reducing concerns about directional pleiotropy.

## 4. Discussion

Our MR analysis found no evidence to support a causal effect of genetically predicted IBD on the risk of VLU. This finding must be interpreted in the context of the existing literature, which consists primarily of case reports and small observational studies [9, 10]. The apparent discrepancy between our null genetic results and these clinical observations is not a contradiction but a clarification. MR estimates the effect of lifelong genetic predisposition, free from confounding by environmental factors or reverse causality. Therefore, our results strongly suggest that the core genetic variants which cause IBD are not the same variants that drive the pathogenesis of VLU.

This crucial distinction allows us to propose a more nuanced model for understanding comorbidities in IBD. The clinical co‐occurrence reported in observations is likely attributable to alternative mechanisms that our genetic approach deliberately circumvents. These include (1) misclassification bias, where ulcers diagnosed as ‘venous’ in IBD patients may in fact be atypical, immune‐mediated lesions such as pyoderma gangrenosum, which share a direct pathological link with IBD [15, 16] and (2) confounding by disease severity and treatment, where patients with more severe, active IBD are exposed to periods of immobility, systemic inflammation, and corticosteroids—all of which are transient, nongenetic risk factors for both disease flares and impaired wound healing. Thus, our study provides the genetic ‘anchor point’ that the observed link is not intrinsic or causal from a genetic standpoint. This reframes the clinical problem from ‘Does IBD cause VLU?’ to ‘What are the specific, nongenetic circumstances under which these conditions co‐occur?’. This is a significant advancement in the conceptual model for gastroenterologists.

While our study clarifies the population‐level genetic relationship, it simultaneously highlights the critical need for next‐generation, phenotypically detailed investigations. The logical next step is to move beyond the question of ‘if’ there is a genetic link to ‘for whom’ and ‘when’ does the clinical risk manifest. This requires (a) individual‐level data integration: future research should leverage large biobanks with linked genetic and electronic health record data to conduct nested MR analyses. This would allow for (a) stratification by IBD disease activity (using clinical and biomarker data), medication exposure (e.g., immunosuppressants), and VLU severity and (b) deep phenotyping of ulcers: genetic studies of VLU would benefit immensely from richer phenotyping, such as distinguishing between ulcers with primary venous insufficiency versus those with significant inflammatory or infectious components. This would help determine if our null finding holds true across all VLU endotypes. By establishing the aggregate genetic baseline, our work sets the stage for these more nuanced analyses that will ultimately deliver personalized clinical insights.

Our study has several limitations that should be considered. First, the generalizability of our findings is constrained by the genetic data source, which is exclusively from European‐ancestry populations. Therefore, our conclusion of a null causal association is primarily valid for European populations and must be validated in future large‐scale GWAS and MR studies involving Asian and other diverse populations. Furthermore, due to data availability, we were unable to perform stratified analyses based on more specific subtypes of lower extremity ulcers (e.g., pyoderma gangrenosum vs. classic venous ulcers), which might have obscured subtype‐specific relationships.

## 5. Conclusion

In conclusion, this MR study found no genetic evidence to support a causal relationship between IBD and VLU. This suggests that the observed clinical co‐occurrence is unlikely to be driven by a shared genetic etiology. Therefore, the management of common venous ulcers in IBD patients should follow standard guidelines and need not be intensified solely based on the IBD diagnosis.

## Ethics Statement

This work does not include any studies performed on humans or animals.

## Consent

The authors have nothing to report.

## Disclosure

All authors read, critically reviewed, and approved the final manuscript as submitted.

## Conflicts of Interest

The authors declare no conflicts of interest.

## Author Contributions

Yanfeng Lin and Xiaohui Qin conceived the topic and draft the manuscript. The revision of manuscript was completed by Jinke Huang and Haiyan Zhang.

## Funding

This work was funded by the National Natural Science Foundation of China (No. 82505455), the Clinical Collaboration Project of Integrated Traditional Chinese and Western Medicine on Major and Complex Diseases: Crohn’s Disease (ZDYN‐2024‐A‐079), and the Specific Research Fund for TCM Science and Technology of Guangdong Province Hospital of Chinese Medicine (No. YN2024GZRPY030).

## Data Availability

All data generated or analyzed during this study are included in the published article.
